# Do withdrawal symptoms predict depression relapse after antidepressant cessation?

**DOI:** 10.1007/s00406-025-02005-z

**Published:** 2025-04-23

**Authors:** Constantin Volkmann, Subati Abulikemu, Isabel M. Berwian, Quentin J. M. Huys, Henrik Walter

**Affiliations:** 1 Department of Psychiatry, Charité-Universitätsmedizin Berlin, Corporate Member of Freie Universität Berlin and Humboldt-Universität zu Berlin, Charité Campus Mitte, 10117 Berlin, Germany; 2 Department of Brain Sciences, Faculty of Medicine, Imperial College London, London, UK; 3 Princeton Neuroscience Institute & Psychology Department, Princeton University, Princeton, USA; 4 Division of Psychiatry, University College London, London, UK; 5 Max Planck UCL Centre for Computational Psychiatry and Ageing Research and Wellcome Centre for Human Neuroimaging, Institute of Neurology, University College London, London, UK

**Keywords:** Antidepressant withdrawal, Depression relapse, Antidepressant discontinuation syndrome, Major depressive disorder

## Abstract

Discontinuing antidepressants after remission poses risks of withdrawal symptoms and relapse. This study addressed four questions: Can withdrawal symptoms be differentiated from relapse? What are their risk factors? Are withdrawal symptoms associated with relapse? Can discontinuation be optimized? 103 patients with a remitted major depressive disorder were randomized to continue or discontinue antidepressants. Withdrawal symptoms were assessed using the Discontinuation Emergent Signs and Symptoms scale (DESS). Withdrawal syndrome was defined as experiencing at least four new or worsened DESS symptoms. Associations between clinical factors and symptom count were examined using linear regressions. After the randomization phase, all patients discontinued treatment and were monitored for six months. The relationship between withdrawal symptoms, clinical factors, and relapse risk was analyzed via logistic regression and a Cox proportional hazards model. Ten symptoms were reported exclusively in the discontinuation group and may aid in distinguishing withdrawal syndrome from relapse. Withdrawal syndrome occurred in 29% (95% PI [8.3%, 72%]) of patients. Women reported more withdrawal symptoms than men (factor 1.67 (95% PI [1.06, 2.56])). None of the other predictors were associated with symptom count. Of 83 patients with outcome data, 54 (65%) remained well and 29 (35%) relapsed. Withdrawal symptoms (0.58, 95% PI [0.07, 1.16]) and early depressive symptoms (0.63, 95% PI [0.16, 1.17]) were associated with a higher relapse risk. Tapering duration was not associated with either withdrawal symptoms or relapse rate. Withdrawal symptoms were common and more frequent in women. Experiencing withdrawal symptoms may increase relapse risk.

**Supplementary Information:**

The online version contains supplementary material available at 10.1007/s00406-025-02005-z.

## Introduction

Antidepressants are used for treating a range of psychiatric conditions, most notably major depressive disorders (MDD). Their global usage has surged in recent years [1], with data indicating that between 2017 and 2018 17% of adults in the UK had an active prescription for these medications [2–4]. Many patients eventually discontinue their antidepressant medication. A recent study found that 42% of users stopped within 2 years of treatment [5]. Reasons for termination of treatment include guidelines recommendations and side effects [6, 7].

Discontinuing antidepressants carries an elevated risk for relapse of depression [8, 9] and can lead to antidepressant withdrawal syndrome. Estimates from meta-analyses of randomized-controlled trials indicate that between 8% an 28% [10, 11] of patients experience withdrawal symptoms, which can range from mild to severe and can last for weeks to months [3, 12–15]. However, these figures vary considerably depending on study design, length of follow-up, and the specific criteria used to define withdrawal. For instance, some analyses that focused on systematic assessment of withdrawal reported incidence rates as high as 44% in the discontinuation arm [11]. Even though the occurrence of adverse effects upon the cessation of antidepressants has been described as early as 1961 [16], research into this issue remains relatively scarce and several uncertainties remain.

In this study, we aimed to address key unanswered questions regarding antidepressant cessation: First, can withdrawal symptoms be differentiated from the re-emergence of depression? And if so, how? Differentiating between withdrawal symptoms and relapse of depression can be challenging due to overlapping symptoms such as mood disturbances, anxiety, and physical complaints. This distinction is crucial because [17–19] misdiagnosing withdrawal symptoms as a relapse of depression may lead to unnecessary continuation or resumption of antidepressant medications, whereas recognizing true relapse ensures timely intervention [17–19].

Second, can the occurrence of withdrawal symptoms be predicted? Patient-level determinants, such as the age at depression onset and female sex, might influence the risk of experiencing such symptoms [20]. Treatment-related factors, such as treatment duration, dosage and type of antidepressant, have been associated with the incidence of ADS [11, 21]. Substances with shorter plasma elimination half-lives, such as paroxetine [22], are associated with a higher risk of withdrawal symptoms [23] and more severe symptoms [24]. Prediction of these symptoms would allow for early clinical intervention to prevent their emergence.

Third, do withdrawal symptoms increase relapse risk? Discontinuation of antidepressants has been linked to an increased relapse risk, particularly when cessation is abrupt [25, 26]. Further research is needed to determine if withdrawal symptoms themselves play a direct role in elevating relapse risk.

Fourth, can the discontinuation process be optimized to reduce the occurrence of withdrawal symptoms? Long-term use of psychotropic medication may establish a new homeostatic equilibrium [27, 28]. Gradual reduction of medication may mitigate withdrawal symptoms by minimizing disruption to this equilibrium [28]. However, high quality evidence supporting gradual tapering is limited [17]. Notably, a recent study found no relationship between abrupt versus slow tapering and the success of discontinuation [29].

To address these questions, we analyzed data from a naturalistic study of antidepressant discontinuation comprising 103 patients.

## Methods

### Ethical approval

for the study was obtained from the cantonal ethics commission Zurich (BASEC: PB_2016-0.01032; KEK-ZH:2014 − 0355) and the Campus Charité-Mitte ethics commission (EA 1/142/14). All procedures followed the Declaration of Helsinki.

### Inclusion criteria

Patients eligible for inclusion had experienced a major depressive episode, achieved remission on antidepressants, and independently decided to discontinue medication prior to study participation. Patients received usual care while participating in the study and provided written informed consent. Detailed inclusion and exclusion criteria are available in Supplementary Table 1.

### Measures

Depressive symptoms were evaluated using both the Inventory of Depressive Symptomatology: Clinician (IDS-C [30]) and Self-report IDS (IDS-S). Trait anxiety was assessed using the State-Trait Anxiety Inventory, Trait Version (STAI-T [31]). Withdrawal symptoms were assessed with the Discontinuation Emergent Signs and Symptom Scale (DESS [24]). The antidepressant withdrawal syndrome was defined as the occurrence of four or more new or worsened symptoms on the DESS scale, based on Rosenbaum’s criteria [24]. Somatic DESS symptoms were defined as symptoms 1 to 14, and psychological symptoms as DESS items 15 to 43. Relapse of major depressive disorder was assessed via clinician-administered diagnostic interviews of the SCID–I [32].

### Study design

**Fig. 1:**
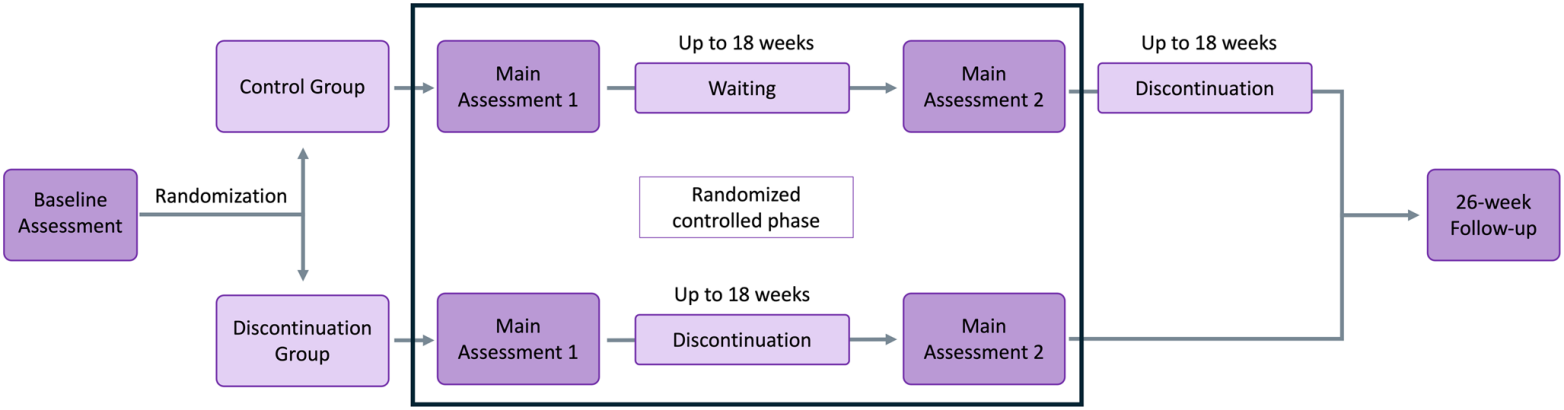
Study design. Patients with a currently remitted major depressive disorder currently taking antidepressant medication underwent a baseline assessment and were then randomized to one of two groups. Patients in the discontinuation group discontinued their medication after main assessment 1, whereas those in the control group waited. Group differences were assessed at main assessment 2, which represents the endpoint of the RCT phase. After discontinuation, all patients entered a six months follow-up. Here, associations between baseline characteristics and post-withdrawal symptoms on the one hand and depression relapse on the other were assessed

We used data from the two-site AIDA trial (Zurich and Berlin) on antidepressant discontinuation [33]. The trial consisted of a randomized-controlled phase and a subsequent 26-week follow-up (Fig. 1). After inclusion, patients underwent a baseline assessment, including patient history and current symptomatology, and were then randomized into either the discontinuation or control group.

Following main assessment 1, which recorded baseline IDS and DESS scores, patients in the discontinuation group began gradual medication tapering over up to 18 weeks, as determined by their treating physicians. There were no restrictions on IDS and DESS assessments were conducted at weeks one, two, and four post-discontinuation. Group differences were evaluated at main assessment 2.

After the randomized-controlled phase, patients in the control group also discontinued their medication, following a similar tapering protocol and IDS/DESS assessment schedule as the discontinuation group. All patients were monitored for six months post-treatment.

### Statistical analyses

All statistical analyses were conducted in R Studio (Version 1.4.1106). We applied both Frequentist and Bayesian methods. Frequentist methods were used when testing a sharp null hypothesis and for sensitivity analyses. Bayesian posterior intervals represent 95% central intervals. Factors were considered “associated” if the 95% intervals did not include zero. Analyses were predefined in an analysis plan (https://github.com/mpc-ucl/aidaShare/tree/master/analysis/DiscontinuationSymptoms).

### Effects of discontinuation

The randomized-controlled phase of the study was used to investigate the effect of antidepressant discontinuation on symptom scores, namely DESS, psychological DESS, somatic DESS, and IDS. We conducted a series of negative binomial regression analyses adjusting for baseline scores. A negative binomial regression with a log link function was chosen due to the over-dispersed nature of the data. For the estimation of a sex-specific effect, we included an interaction between group and sex. Estimates were back-transformed using an exponential function to provide more interpretable results, representing the multiplicative effect of the predictors on the outcome.

Group differences at main assessment 2 were used to identify specific DESS symptoms associated with antidepressant discontinuation. p-values were generated using Fisher’s Exact Test.

### Disentangling depression and withdrawal symptoms

#### Temporal course of IDS and DESS

To compare the trajectories of DESS and IDS symptoms, we conducted two sets of analyses. First, we assessed the timing of each patient’s maximum DESS or IDS score using a paired Wilcoxon rank-sum test. Next, we compared maxima of quadratic regressions fitted to the DESS and IDS values. We included a random intercept for the factor patient in a mixed-effects model. Pre-discontinuation values served as baseline, with time set to 0. For all analyses of symptom severity, DESS and IDS scores were modelled using a negative-binomial link function. For sensitivity analyses, a normal link function in a Bayesian and a Frequentist framework was used.

#### Cross-lagged panel model

A Cross-lagged panel model was fitted to examine how symptoms influenced each other over time, particularly to disentangle withdrawal symptoms from depressive symptoms. We used Full Information Maximum Likelihood (FIML) estimation to handle missing data, thus including all available observations in the model and minimizing bias. The analyses were conducted using the ‘lavaan’ package in R. The analysis was repeated using DESS symptoms that were significantly elevated after discontinuation.

#### Assessing misdiagnosis of withdrawal as relapse

To examine whether withdrawal-related symptoms might have been misdiagnosed as relapse, we compared the proportion of patients with any matching IDS symptoms between the withdrawal phase and at the final assessment at study exit. We assessed whether this proportion differed between patients who experienced a relapse and those who did not, using Pearson’s Chi-squared test and Fisher’s exact test.

#### Association of medical factors with withdrawal symptoms

We assessed the association between clinical factors and withdrawal symptoms using data from all patients. The predefined predictor variables included tapering duration, drug half-life, treatment duration, medication load, trait anxiety, sex, age at disease onset, and the interaction between tapering duration and drug half-life. Medication load was calculated as dosage prior to discontinuation divided by the maximal allowed dose according to the Swiss compendium (www.compendium.ch), adjusted for participant weight.

To estimate symptom severity by antidepressant class and type, we used a varying intercept model with partial pooling, allowing the intercept for each antidepressant to vary within its class. We defined antidepressant classes as follows:

1. Selective serotonin reuptake inhibitors (SSRIs): Citalopram, Escitalopram, Fluoxetine, Paroxetine, Sertraline, Vortioxetine.
2. Serotonin and norepinephrine reuptake inhibitors (SNRIs): Duloxetine, Venlafaxine.
3. Dopamine reuptake inhibitors (DNRIs): Bupropion.
4. Other: Agomelatine.

To test the predictive value of clinical characteristics regarding the severity of antidepressant withdrawal, we conducted a multiple regression using Zurich as a training sample and Berlin as a hold-out sample. We calculated the Mean Absolute Error (MAE), which refers to the average error between predicted and observed values. The MAE was compared to the Mean Absolute Deviation (MAD), which refers to the mean deviation of values from the sample median.

#### Association of withdrawal symptoms with relapse risk

We used logistic regression to examine whether withdrawal symptoms act as a stressor for relapse. Since trait anxiety (STAI-T) might contribute to both withdrawal symptoms and relapse, we included it as a predictor in the multiple regression. We conducted a univariate Cox proportional hazards regression to relate DESS values to time-to-relapse. We averaged DESS values across all three post-discontinuation assessments. To ensure relapse risk was related to withdrawal symptoms arising from antidepressant withdrawal and not trait anxiety, we controlled for baseline STAI-T. We addressed missing relapse values using best-worst imputation.

#### Relapse prediction

We explored the possibility of deriving a clinically informative relapse prediction model from clinical characteristics. We used the Zurich sample as the training set to fit a logistic regression model, with average post-withdrawal DESS scores, STAI-T, and their interaction as predictors. We evaluated the model’s performance using 10-fold cross-validation with 10 repetitions, resampling at each iteration. We evaluated the predictive power of the logistic model using the Berlin data as an external validation set. Logistic regression weights from the Zurich sample were used to predict the relapse status of Berlin patients. Since the Berlin sample was included in previous analyses, it cannot be considered as an entirely independent dataset. Therefore, this analysis served as a check for statistical generalizability.

## Results

### Sample description

103 patients were included in the study. 50 and 53 were randomized to the discontinuation group and the control group, respectively. Demographics and treatment characteristics were comparable across the groups (Table 1). 14 patients dropped out and 1 relapsed before any post-discontinuation assessment and were thus excluded from analyses. Among the included patients, 54 (65%) remained well and 29 (35%) relapsed (missing data for 5 patients). Supplementary Table 2 shows the frequency of antidepressants used and the respective percentage of patients not completing the discontinuation phase, which varied significantly between antidepressants (*p* < 0.01). Treatment duration, dosage levels and discontinuation speed were not associated with higher drop-outs (supplementary material). Supplementary Fig. 1 shows the distribution of treatment and tapering duration.

**Table 1:** Demographic, clinical and medication related variables at baseline

	Control Group (*N* = 53)	Discontinuation Group (*N* = 50)	No relapse (*N* = 54)	Relapse (*N* = 30)	*P* value
Age	36.7 (11.5)	33.06 (10.49)	33.56 (11.23)	37 (10.95)	0.2
Male sex, No (%)	14 (26.4)	10 (20.0)	13 (24.1)	7 (23.3)	1
No of episodes	2.77 (1.42)	1.94 (1.13)	2.22 (1.31)	2.6 (1.25)	0.2
Age of depression onset	22.91 (8.48)	23.2 (8.84)	22.24 (7.86)	24.9 (9.88)	0.2
Residual depression	4.25 (3.64)	4.0 (3.26)	3.81 (3.36)	3.7 (1.31)	0.9
Treatment duration (months)	30.09 (29.41)	37.19 (34.65)	32.56 (29.93)	36.78 (37.82)	0.6
Tapering duration (days)	46.07 (42.12)	52.77 (35.77)	48.89 (39.79)	51.1 (40.64)	0.8

Mean and SD values unless stated otherwise. Residual depression: IDS score at baseline. P values refer to the difference between relapse and no relapse

### Effects of antidepressant withdrawal on symptom scores

**Fig. 2:**
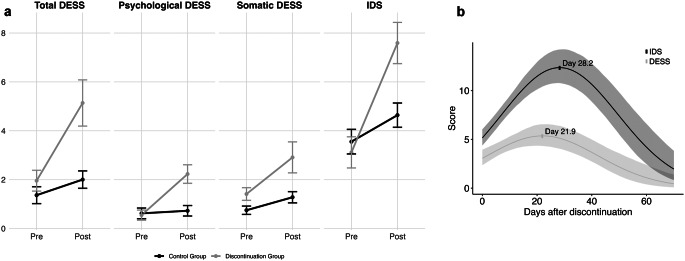
**(a)** Effect of discontinuation on symptom scores. Baseline and endpoint means of total, psychological, somatic DESS symptoms and IDS. MA1 = main assessment 1, MA2 = main assessment 2. **(b)** Temporal course of DESS and IDS: Lines represent the quadratic fits with a negative binomial link function; marking ticks represent time values (x-axis) corresponding to maximum instrument scores (y-axis). Shaded areas indicate 95% posterior intervals of means

Patients in the discontinuation group reported significantly more DESS symptoms (Estimate = 2.53, 95% PI [1.48, 4.21]) and IDS symptoms (Estimate = 1.68, 95% PI [1.24, 2.25]) compared to the control group. This increase was observed in both psychological (Estimate = 3.32, 95% PI [1.69, 6.70]) and somatic DESS symptoms (Estimate = 1.97, 95% PI [1.10, 3.62]). Results of Frequentist regressions are shown in Supplementary Table 3. Figure 2a shows mean and standard errors of DESS and IDS scores across groups. Psychological and somatic DESS symptoms were strongly correlated (Pearson’s *r* = 0.73, *p* = 2.6*10^− 8^).

In the discontinuation group, 50% of patients reported experiencing four or more DESS symptoms, compared to 21% in the control group. Based on these results, the overall incidence rate of antidepressant withdrawal syndrome was estimated to be 29% (95% PI [8.3%, 72%]). Earlier assessments were associated with more symptoms (Factor time (days) = 0.95, 95% PI [0.92, 0.98]), suggesting that symptom load decreases over time.

Withdrawal symptoms increased in response to discontinuation in women (MD = 3.84, 95% PI [1.26, 8.59]), while remaining comparable between the groups in men (MD = 0.20, 95% PI [-0.95, 3.80]).

### Prevalence of specific symptoms

The most frequently reported symptoms were dizziness (31.8% in the discontinuation group vs. 4.3% in controls) and irritability (38.6 vs. 12.8%). Nine symptoms occurred significantly more often in the withdrawal group (based on uncorrected p values of Fisher’s exact test). Additionally, ten symptoms were reported exclusively in the withdrawal group, nine of which were physical in nature (Supplementary Table 4). Overall, 20.5% of patients in the discontinuation group reported at least one of these specific symptoms.

### Distinguishing depressive symptoms from withdrawal effects

Maximum DESS scores were reached earlier than maximum IDS scores after discontinuation (Paired Wilcoxon rank-sum test: MD = − 5.6 days, Z=-3.5, *p* = 0.001). When fitting a quadratic function to the time course of DESS and IDS scores, the maximum DESS scores occurred on day 21.9, whereas maximum IDS scores occurred on day 28.2 (Fig. 2b).

IDS and DESS scores were correlated at weeks 1,2 and 4 (Coefficient of correlation = 0.74, 0.66 and 0.61, respectively) and the cross-lagged panel model fit the data adequately (χ²(12) = 15.528, *p* = 0.214). Withdrawal symptoms did not predict subsequent withdrawal symptoms better than depressive symptoms did. Significant covariation between withdrawal and depressive symptoms was observed at week 1 (β = 49.982, *p* < 0.01) and week 4 (β = 22.747, *p* < 0.001). The strong covariation suggests shared features, making it challenging to disentangle these symptoms in clinical settings. We repeated the analysis using only the ten DESS symptoms that occurred exclusively in the discontinuation group. The fit was worse compared to the full model (χ²(12) = 22.324, *p* = 0.034), and the covariation between depressive and withdrawal symptoms remained significant at week 1 (β = 1.332, *p* = 0.001) but not significant at week 4 (β = 1.362, *p* = 0.160).

The proportion of patients with matching symptoms during withdrawal and at study exit was similar between relapsed (43.3%) and non-relapsed (38.9%) patients. Neither Pearson’s Chi-squared test (*p* = 0.868) nor Fisher’s exact test (*p* = 0.817) showed significant differences between the groups. These findings suggest that withdrawal symptoms were not systematically misdiagnosed as relapse.

### Clinical factors associated with withdrawal symptoms

**Fig. 3:**
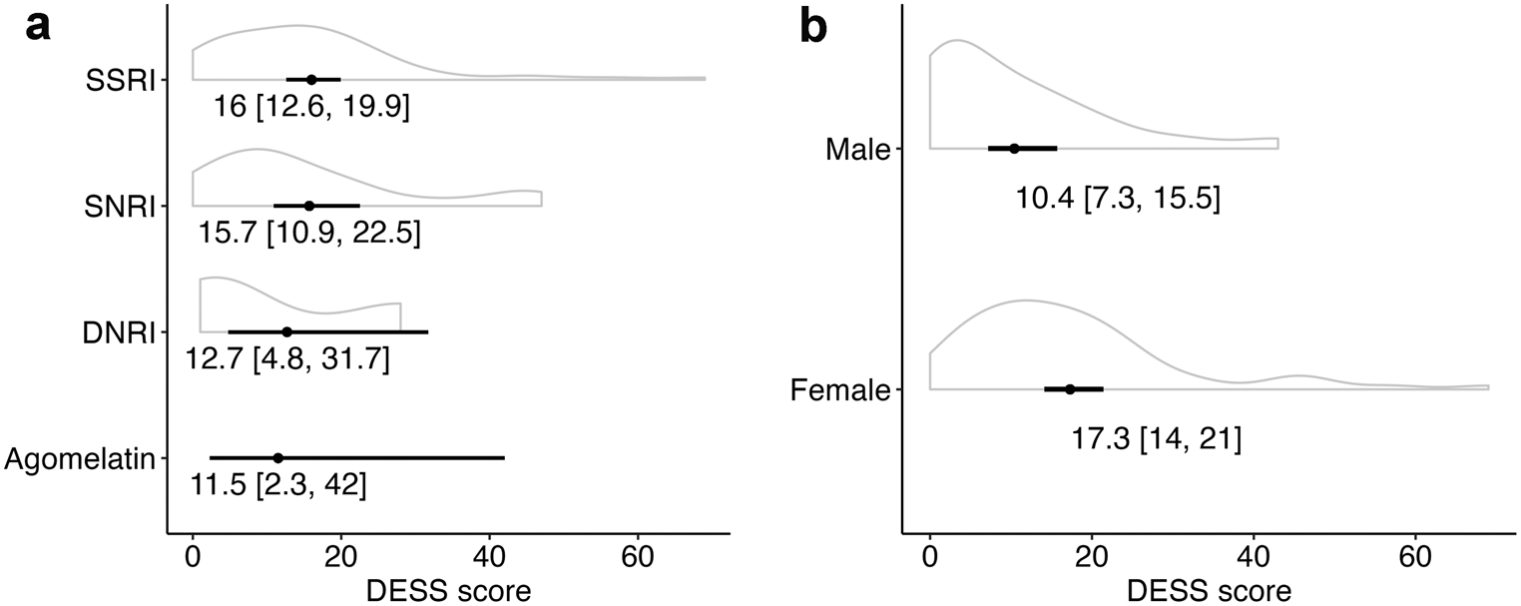
DESS symptom count by antidepressant class and sex. Violin plots showing total number of DESS symptoms after antidepressant discontinuation for different antidepressant classes and by sex. Posterior means and 95% intervals (Black dots and lines) were estimated in a negative-binomial regression with partial pooling. The outcome was the sum of DESS symptoms at weeks 1,2 and 4. Missing values were imputed using last-observation-carried-forward (LOCF)

For the following analyses, data from the 26-week follow-up period were considered, during which patients from both groups had discontinued their medication. Among predefined predictors, only sex demonstrated an association with the number of DESS symptoms (Table 2; Fig. 3). This relationship persisted when including trait anxiety and its interaction with sex as predictors (Supplementary Fig. 3). Slower discontinuation was not associated with fewer symptoms. Sensitivity analysis using different priors produced similar results (Supplementary Table 5).

**Table 2:** Association between clinical features and number of DESS symptoms

	Bayesian Mean and 95% interval	Confidence interval	*p* value	FDR *p* value
Tapering duration (months)	1.10 [0.98, 1.27]	[0.97, 1.25]	0.17	0.58
Drug half-life (days)	1.07 [0.90, 1.30]	[0.74, 1.44]	0.52	0.78
Duration of treatment (years)	1.00 [0.94, 1.08]	[0.94, 1.08]	0.96	0.96
Medication load (z- score)	1.04 [0.85, 1.29]	[0.85, 1.29]	0.67	0.78
Age of illness onset (decades)	0.68 [0.11, 5.23]	[0.11, 4.17]	0.65	0.78
Sex (female)	1.67 [1.06, 2.56]	[1.08, 2.54]	0.018	0.12
STAI-T (z-score)	1.09 [0.92, 1.31]	[0.91, 1.30]	0.34	0.78

Posterior mean and 95% intervals and frequentist confidence intervals for univariate regressions with different predictors. Total number of DESS symptoms (sum of weeks 1, 2 and 4) as outcome with negative-binomial link function. Coefficients are multiplicative, meaning that for each unit increase in predictor value, the factor slope multiplies the outcome

Incorporating all factors that we hypothesized to be relevant, our model did not successfully predict the severity of withdrawal symptoms in an out-of-sample test, where Berlin served as a hold-out sample. The Mean Absolute Error (MAE) of predicted versus actual DESS values was greater than the Mean Absolute Deviation (MAD) of the Berlin sample (14.99 versus 10.55). This means that the prediction model was less accurate than a simple benchmark prediction based on the sample median (Supplementary Fig. 4).

### Type of antidepressant and withdrawal symptoms

Withdrawal symptoms were numerically higher for SSRIs and SNRIs than for Bupropion and Agomelatine (Fig. 3). Due to the small sample sizes for Agomelatine (*n* = 1) and Bupropion (*n* = 4), no statistical inferences could be drawn. Supplementary Fig. 5 shows results for each SSRI.

### Association of clinical factors with relapse of depression

DESS and IDS scores were individually positively associated with relapse risk, while no association was detected for sex and trait anxiety (Table 3). Including STAI-T, as well as the interaction of DESS and STAI-T as predictors did not change the association of DESS scores and relapse risk. Restricting DESS symptoms to early (first two weeks) or somatic symptoms yielded similar, while less certain, results (Supplementary Tables 6 and 7). Of note, duration of treatment and tapering duration showed no association with relapse risk. Including an interaction between DESS and tapering duration did not change these results.

**Table 3:** Association between clinical features and relapse of depression

Variable	Bayesian Interval	Risk Difference (%)	*P* value	FDR *p* value
DESS	0.58 [0.07, 1.16]	13.9 [1.7, 28.1]	0.04	0.11
Sex	-0.07 [-1.09, 0.98]	-0.2 [-17.8, 20.3]	0.94	0.94
STAI-T	0.42 [-0.02, 0.90]	10.1 [-0.2, 21.9]	0.06	0.13
IDS	0.63 [0.16, 1.17]	15.2 [3.8, 28.3]	0.01	0.08
Tapering duration	0.05 [-0.42, 0.51]	1.0 [-9.4, 12.0]	0.81	0.94
Treatment duration	0.12 [-0.33, 0.57]	3.0 [-7.6, 14.0]	0.57	0.86
**Multiple regression** **Predictor variable**		**Bayesian Interval**		
DESS		0.60 [0.08, 1.25]		
STAI-T		0.41 [-0.07, 0.94]		
Interaction		-0.37 [-0.91, 0.14]		
**Multiple regression** **Predictor variable**		**Bayesian Interval**		
DESS		0.63 [0.09, 1.23]		
Tapering duration		-0.05 [-0.65, 0.49]		
Interaction		0.25 [-0.15, 0.94]		

Risk difference refers to the absolute increase in relapse risk for 1 unit (SD) increase in predictor value. Sex: female = 0, male = 1. Brackets represent 95% bayesian posterior intervals. P values correspond to frequentist binomial regression. IDS and DESS are mean of three post withdrawal assessments (weeks 1, 2 and 4). Predictors were z-scored for better interpretability. Complete case analysis. The table below depicts results of two multiple regression analyses

We conducted best-worst, as well as multiple random imputations to account for missing data regarding relapse status. 32, 66 and 100% of intervals with random imputations were credibly positive for STAI-T, DESS and IDS, respectively (Supplementary Tables 8 and 9).

We repeated the logistic regression using the average sum score of ten discontinuation-specific symptoms across post-discontinuation assessments as the predictor. Although the certainty of the association decreased, a positive relationship persisted (estimate = 0.50, 95% PI [-0.03, 1.10]). The results are summarized in Supplementary Table 8.

**Fig. 4:**
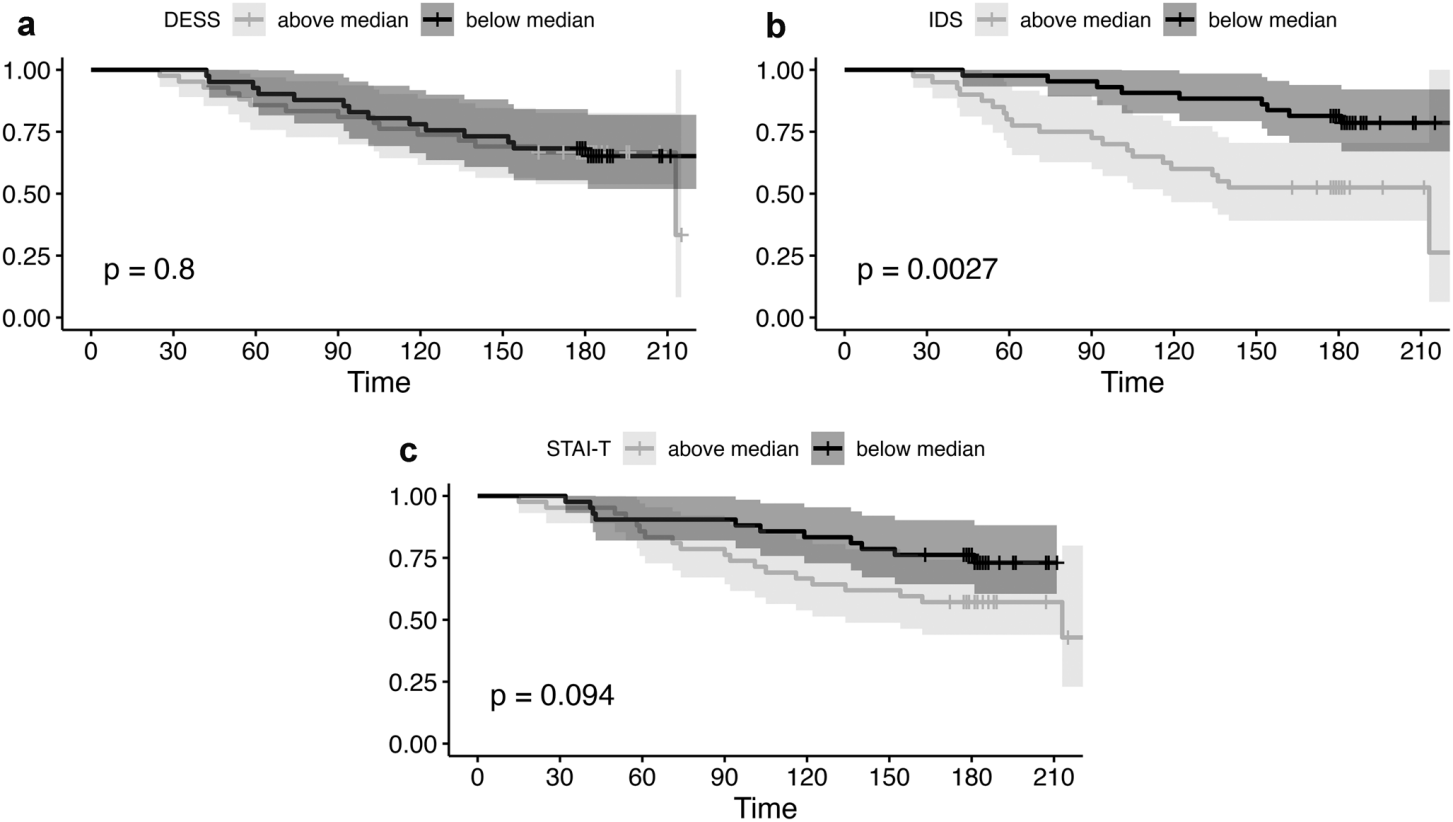
Kaplan-Meier curves for relapse of a depressive episode. Univariate analysis with post-discontinuation average scores of DESS (**a**), IDS (**b**) and STAI (**c**) as predictors. Strata were generated via median-split

In the proportional hazards model, both DESS and IDS showed a significant association with relapse, as detailed in Table 4. In contrast, no significant association was seen for STAI-T. The positive associations remained, when controlling for age, sex, and STAI-T and when missing relapse values were addressed through best-worst imputation (Supplementary Tables 10 and 11). Excluding patients who relapsed within 30 days (potentially misinterpreted withdrawal symptoms) did not change the results for IDS (1.44 [1.08, 1.95]), while the association became less certain for DESS (1.31 [0.98, 1.72]). Kaplan-Meier curves were not significant for DESS (Fig. 4), as median-split leads to information loss.

**Table 4:** Relapse survival analysis. Results of univariate Cox regressions

	HR (95% Interval)	*p*-value	FDR *p*-value
Average DESS	1.35 [1.03, 1.78]	0.032	0.07
DESS (< 14 days)	1.25 [0.87, 1.79]	0.22	0.28
DESS (day 15–30)	1.36 [1.01, 1.89]	0.046	0.08
Average IDS	1.49 [1.12, 1.98]	0.006	0.03
IDS (< 14 days)	1.66 [1.18, 2.32]	0.0037	0.02
IDS (days 15–30)	1.53 [1.15, 2.05]	0.0037	0.02
Sex	1.09 [0.46, 2.54]	0.85	0.92
STAI-T	1.31 [0.96, 1.80]	0.087	0.15
Age of depression onset	1.19 [0.87, 1.62]	0.28	0.33

Results of Frequentist Cox regressions. Predictors were z-scored for better interpretability. Hazard ratio (HR) signifies change in relapse risk for each SD increase in predictor value. Complete case analysis

### Relapse prediction

The multiple logistic regression model including DESS and STAIT-T correctly classified 521 out of 830 resampled observations (balanced accuracy = 0.52). Specifically, 48 out of 290 relapsed and 437 out of 540 non-relapsed observations were correctly predicted (sensitivity = 0.17, specificity = 0.88). The area under the ROC curve (AUC) was 0.6, not suggestive of an acceptable performance. The out-of-sample prediction correctly classified the relapse-state of 15 out of 24 patients with a balanced accuracy of 0.56 and an AUC of 0.67. The model correctly classified 1 out of 9 relapsed (sensitivity = 0.11) and 14 out of 15 non-relapsed patients (specificity = 0.93). In summary, no acceptable predictive performance was achieved.

## Discussion

In this observational trial, 103 patients with remitted major depressive disorder gradually tapered off antidepressant medication over an average of 50 days. Overall, 65% of patients maintained remission after completing antidepressant discontinuation, while 35% experienced a relapse. This relatively high relapse rate is consistent with prior literature indicating an elevated relapse risk following cessation of antidepressants [8]. The incidence of antidepressant withdrawal syndrome, defined as four or more DESS symptoms, was 29%. This incidence is higher than the rates reported in two recent meta-analyses [10–14], which found 8% and 28% in RCTs, respectively [10, 11]. The open label design might explain some of the observed discrepancies, as it may have influenced patient expectations and symptom reporting. Importantly, the average duration of treatment in our trial was considerably longer than in RCTs, which could have contributed to the higher incidence of withdrawal symptoms.

Our results suggest that total symptom scores are insufficient as a diagnostic tool and for differentiating between withdrawal symptoms and depressive relapse. Notably, 20% of patients met the symptom-level criterion of antidepressant withdrawal syndrome before beginning discontinuation. Furthermore, the IDS and DESS scores were highly correlated, indicating a substantial overlap between depressive symptoms and withdrawal symptoms, and both were significantly associated with relapse.

Differentiation between withdrawal and relapse may be achieved through a combination of factors. One notable finding is the different time course of symptom evolution, with DESS scores peaking earlier than IDS scores, which may aid in distinguishing between the two [17, 19].

Our results support previous reports [34] that certain somatic symptoms may help identify withdrawal syndrome. Ten symptoms, such as incoordination, blurred vision and shaking, occurred exclusively after antidepressant discontinuation.

The cross-lagged panel model further suggests that using a reduced set of symptoms may help reduce the overlap with depressive symptoms. The weaker covariation observed with the subset of discontinuation-specific symptoms indicates some improvement in differentiation, though the worse model fit underscores the limitations of using a smaller set of symptoms for this purpose.

Therefore, differential diagnosis should consider both time course and the occurrence of specific symptoms, rather than total symptom scores alone. Moreover, clinical observations indicate that withdrawal symptoms tend to resolve after reintroducing or increasing the dose of the antidepressant and are typically of a more transient nature compared to a depressive relapse [17]. This difference in symptom duration and treatment response can further aid in distinguishing between withdrawal and relapse.

We were unable to derive a clinically useful prediction model regarding withdrawal symptoms. Individual risk factors may yet be useful in identifying patients at risk. Consistent with previous publications [11, 20], we found that women experience more withdrawal symptoms than men. Interestingly, no discernible difference in DESS scores between the withdrawal and the control group was found for men. More studies are needed before drawing firm conclusions regarding a sex-specific effect.

Our findings suggest that the emergence of withdrawal symptoms, particularly early depressive symptoms after antidepressant discontinuation, may increase the risk for subsequent depression relapse. Restricting the analysis to withdrawal-specific symptoms did not significantly change this association. It remains unclear whether withdrawal symptoms and relapse are causally related or are independent consequences of antidepressant discontinuation. Alternatively, some withdrawal symptoms may be misinterpreted as relapse [35]. However, the persistence of a positive relationship after excluding patients who relapsed within the first month argues against the misdiagnosis hypothesis. If withdrawal symptoms were commonly mistaken for relapse, we would expect these relapses to occur predominantly early in the discontinuation process when withdrawal symptoms peak. Furthermore, individual depressive symptoms present at study exit were not more frequently observed during the withdrawal phase in the relapsed versus in the non-relapsed group. Nevertheless, we acknowledge that disentangling withdrawal from true relapse is challenging, as some researchers suggest that many cases labeled as recurrence could represent persistent post-withdrawal syndromes [36]. Therefore, while our data do not strongly support the notion that withdrawal symptoms were misinterpreted as relapse, we cannot exclude this possibility [19].

Additionally, while evidence suggests that individuals with comorbid anxiety disorders have higher relapse rates [37, 38], our study did not find a significant association between trait anxiety and relapse.

We found no association between treatment duration, dosage level, or antidepressant plasma half-life and discontinuation-emergent events. This is in line with recent meta-analyses that did not find significant associations for these predictors [11]. Interestingly, a longer tapering duration showed a non-significant trend toward being associated with more withdrawal symptoms, rather than fewer, and was not associated with relapse risk. As discontinuation speed was not controlled by the study but was determined by treating physicians, this association could be due to reverse causation, where more symptoms may have led to slower tapering. Alternatively, an influence of tapering duration on withdrawal symptom severity may only be observable with longer tapering periods [28]. It is also possible that no such relationship exists. The impact of tapering speed on withdrawal symptoms remains unclear [19] and should be investigated in randomized-controlled trials.

### Strength and limitation

A key limitation is the open label and observational nature of the trial, which may have led to nocebo effects or biased symptom reporting and affected the association with depression relapse. No information on the diagnosis regarding antidepressant withdrawal syndrome was available, as the study focused on symptoms rather than diagnostic status. The Chouinard criteria [39], which provide a more standardized approach to diagnosing antidepressant withdrawal syndrome, were published after the study had started and therefore could not be used as a diagnostic tool during the trial. Additionally, withdrawal symptoms were not systematically assessed during the tapering phase, which could last up to 18 weeks, potentially missing important symptom patterns during this period. No definitive conclusions regarding the optimal tapering schedules could be drawn, because our data did not include detailed information beyond tapering duration and initial dose (e.g. hyperbolic versus linear reductions). The small sample size and limited number of predictor variables may have hindered the development of an accurate and clinically useful prediction model [40]. Lastly, the exclusion of patients that dropped out before completing discontinuation may have led to an underestimation of withdrawal symptom severity, as some patients might have dropped out due to discontinuation-related symptoms. Because withdrawal symptoms were only assessed after completion, we were unable to determine whether more severe withdrawal symptoms are associated with discontinuation success.

The strengths of this study, however, are notable. First, the long average treatment duration and the use of a control condition combines the advantages of observational trials and RCTs. Second, the study’s real-world outpatient setting and the use of individualized tapering regimens provides a more naturalistic view of antidepressant discontinuation, which can differ significantly from controlled clinical trial conditions. Third, withdrawal symptoms were assessed over a course of 4 weeks – long enough for most antidepressants to be eliminated from the body – thereby reducing the risk of underestimating symptoms that can appear late and persist over several weeks [13].

### Future directions and clinical implications

The significant correlation between withdrawal and depressive symptoms highlights the potential for misinterpreting withdrawal symptoms as depression relapse, or vice versa. The low specificity of the DESS scale implies a potential for false positive diagnoses of withdrawal symptoms. Focusing on more specific withdrawal symptoms and their time course might improve diagnostic precision. In future research, factor analysis with a large sample size should be applied to identify specific symptom clusters and trajectories. To differentiate withdrawal symptoms from depressive relapse, future trials should compare discontinuation-related symptom patterns with those observed during depressive relapse in medication-naïve cohorts. Future studies should focus on identifying patient-specific risk factors for withdrawal symptoms, as this could help identify individuals at risk and allow for tailored intervention strategies.

Clinicians should be aware that experiencing discontinuation-emergent symptoms might pose a risk factor for a depressive relapse. It remains unclear, however, if slower tapering can reduce this risk [10]. A recent publication on antipsychotic discontinuation found no relationship between discontinuation rate and relapse of psychotic disorders [41]. Randomized controlled trials should investigate whether slower tapering can mitigate withdrawal symptoms and the increased relapse rate. They should be conducted in a double-blind fashion to minimize the influence of nocebo effects. Importantly, patients should be informed of the possibility of discontinuation-emergent events before initiation of treatment with antidepressants.

## Electronic supplementary material

Below is the link to the electronic supplementary material.

Supplementary Material 1
