# Supplementary material for: Do withdrawal symptoms predict depression relapse after antidepressant cessation?
Source: Eur Arch Psychiatry Clin Neurosci. 2025 Apr 23;276(2):749–60. doi: 10.1007/s00406-025-02005-z (PMC12953489; doi:10.1007/s00406-025-02005-z)
Supplement: Supplementary file 1 — Supplementary Material 1 [file 406_2025_2005_MOESM1_ESM.docx]

# **Do withdrawal symptoms predict relapse after antidepressant cessation? - Supplementary Material**

## Inclusion and exclusion criteria

Participants fulfilling the following inclusion criteria were eligible for participation in the study:

1. age 18-55 years

2. ability to consent and adhere to the study protocol

3. written informed consent

4. fluent in written and spoken German.

Patients had to additionally fulfil the following criteria:

1. currently under medical care with a psychiatrist or general practitioner for remitted Major Depressive Disorder

and willing to remain in care for the duration of the study (approx. 9 months)

2. informed choice to discontinue medication (including willingness to taper the medication over at most 12 weeks) that was independent of study participation

3. clinical remission (HAMD17 of less than 7) had been achieved under therapy with Antidepressant Medication without having undergone manualized psychotherapy; with no other concurrent psychotropic

medication and had been maintained for a minimum of 30 days,

4. consent to information exchange between treating physician and study team members regarding inclusion/exclusion criteria and past medical history.

Any of the following exclusion criteria led to exclusion of a participant. This included the following general criteria

1. any disease of type and severity sufficient to influence the planned measurement or to interfere with the

parameters of interest (This includes neurological, endocrinological, oncological comorbidities, a history of

traumatic or other brain injury, neurosurgery or longer loss of consciousness.)

2. premenstrual syndrome (ICD-10 N94.3).

and MRI-related criteria

1. MRI-incompatible metal parts in the body,

2. inability to sit or lie still for a longer period,

3. possibility of presence of any metal fragments in the body,

4. pregnancy,

5. pacemaker, neurostimulator or any other head or heart implants,

6. claustrophobia and

7. dependence on hearing aid.

For patients the following additional criteria would lead to exclusion:

1. current psychotropic medication other than antidepressants,

2. questionable history of major depressive episodes without complicating factors,

3. current acute suicidality,

4. lifetime or current axis II diagnosis of borderline or antisocial personality disorder,

5. lifetime or current psychotic disorder of any kind, bipolar disorder,

6. current posttraumatic stress disorder, obsessive compulsive disorder, or eating disorder

7. current drug use disorder (with the exception of nicotine) or within the past 5 years.

***Supplementary table 1. Inclusion and exclusion criteria.***

## Type of antidepressants and dropout rates

The proportion of patients that dropped out during antidepressant discontinuation varied between antidepressant types. The median chi-squared p-value from 1000 simulations was 0.005, with a maximum value of 0.011. The highest dropout rates were observed for Paroxetine (2 of 3), Duloxetine (3 of 6) and Sertraline (4 of 11. Supplementary table 2). Duration of treatment (T-test p-value = 0.88), discontinuation speed (p = 0.36) and dosage level (p = 0.20) did not differ significantly between drop-outs and patients who completed discontinuation.

| **Antidepressant N Dropout (N) Dropout (%)** |
| --- |

Agomelatine 1 0 0

Bupropion 4 1 25

Citalopram 17 1 6

Duloxetine 6 3 50

Escitalopram 35 2 6

Fluoxetine 4 0 0

Paroxetine 3 2 67

Sertraline 11 4 36

Venlafaxine 21 2 10

Vortioxetine 1 0 0

***Supplementary table 2. Frequencies and dropout rates per antidepressant type.***

## Treatment and tapering duration

The average treatment duration was 33.5 months (SD = 32.1; range 3–168) and the average tapering duration was 49.5 days (SD = 39.0; range 0–216). Figure 1 shows the distribution of treatment duration in months and tapering duration in days.


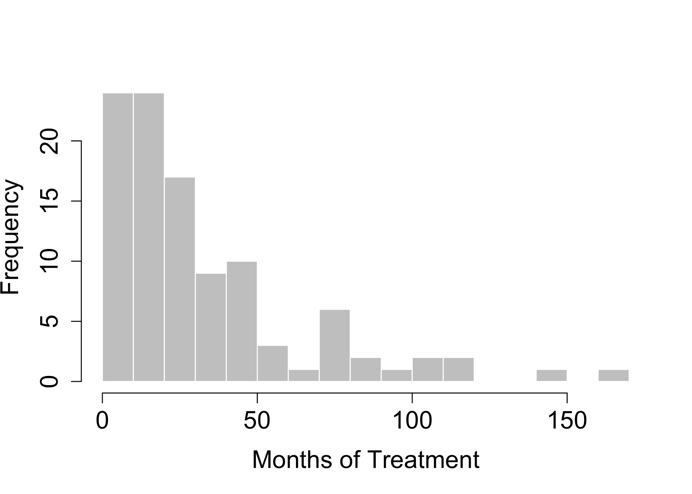

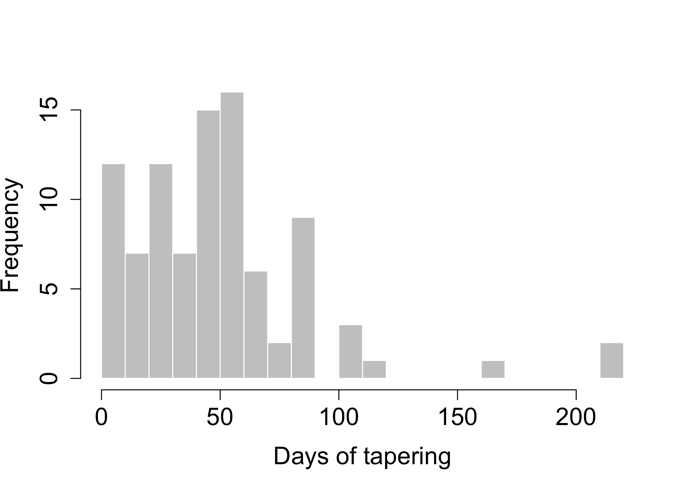


***Supplementary figure 1. Histogram of treatment duration and tapering duration.***

## Effects of antidepressant withdrawal on symptom scores

We modeled DESS_MA2_ using a negative-binomial distribution to account for count data that are overdispersed. We used vaguely informative priors to avoid overfitting the model while still incorporating prior beliefs.

DESS_MA2_ ~ negative−binomial (μ, scale)

log(μ) ~ α + β * DESS_MA1_ + γ * group(0,1)

Vaguely informative priors:

α ~ norm(0,5)

β,γ, 𝝳 ~ norm(0,1)

scale ~ cauchy(0,2)

Categorical antidepressant discontinuation syndrome (ADS):

ADS ~ binom(1,p)

Log(p) ~ group(0,1)

**Outcome Estimate [CI] p-value FDR p-value**

Total DESS 2.53 [1.54, 4.17] < 0.001 < 0.001

Psychological DESS 3.27 [1.71, 6.24] < 0.001 < 0.001

Somatic DESS 1.97[1.11, 3.49] 0.020 0.020

IDS 1.67 [1.25, 2.22] < 0.001 < 0.001

***Supplementary table 3. Effect of discontinuation on symptom scores.*** *Results of Frequentist negative binomial regression with baseline correction comparing discontinuation and control group. Results were back-transformed for better interpretability. CI: confidence interval.*

| **Symptom** | **Withdrawal (%)** | **Control (%)** | **Difference**  **Absolute** | **(%)**  **Relative** | **p-value** | **FDR** |  |
| --- | --- | --- | --- | --- | --- | --- | --- |
| Dizziness, lightheadedness,  or sensation of spinning (vertigo) | 31.8 | 4.3 | 27.6 | 88.2 | 0.001 | 0.028 | |
| Irritability | 38.6 | 12.8 | 25.9 | 75.2 | 0.007 | 0.065 | |
| Bouts of crying or tearfulness | 29.5 | 6.4 | 23.2 | 82.2 | 0.005 | 0.065 | |
| Mood swings | 27.3 | 6.4 | 20.9 | 81 | 0.01 | 0.065 | |
| Trouble sleeping, Insomnia | 20.5 | 2.1 | 18.3 | 90.6 | 0.006 | 0.065 | |
| Headache | 22.7 | 8.5 | 14.2 | 72.8 | 0.082 | 0.300 | |
| Unsteady gait or incoordination | 13.6 | 0.0 | 13.6 | 100 | 0.011 | 0.065 | |
| Blurred vision | 13.6 | 0.0 | 13.6 | 100 | 0.011 | 0.065 | |
| Confusion or trouble concentrating | 18.2 | 6.4 | 11.8 | 74 | 0.110 | 0.301 | |
| Ringing or noises in the ears | 15.9 | 4.3 | 11.7 | 78.9 | 0.084 | 0.300 | |
| Shaking, trembling | 11.4 | 0.0 | 11.4 | 100 | 0.023 | 0.112 | |
| Chills | 11.4 | 0.0 | 11.4 | 100 | 0.023 | 0.112 | |
| Nervousness or anxiety | 13.6 | 4.3 | 9.4 | 76.2 | 0.15 | 0.357 | |
| Unusual sensitivity to sound | 13.6 | 4.3 | 9.4 | 76.2 | 0.15 | 0.357 | |
| Sudden outbursts of anger  (“anger attacks”) | 11.4 | 2.1 | 9.2 | 84.2 | 0.10 | 0.301 | |
| Elevated mood, feeling high | 9.1 | 0.0 | 9.1 | 100 | 0.051 | 0.218 | |
| Sudden worsening of mood | 18.2 | 10.6 | 7.5 | 63.1 | 0.38 | 0.734 | |
| Nose running | 11.4 | 4.3 | 7.1 | 72.8 | 0.26 | 0.552 | |
| Sore eyes | 9.1 | 2.1 | 7.0 | 81 | 0.19 | 0.438 | |
| Problems with speech or speaking clearly | 6.8 | 0.0 | 6.8 | 100 | 0.11 | 0.301 | |
| Unusual visual sensations (light, colors, geometric shapes) | 6.8 | 0.0 | 6.8 | 100 | 0.110 | 0.301 | |
| Fatigue, tiredness | 29.5 | 23.4 | 6.1 | 55.8 | 0.63 | 0.848 | |
| Increased dreaming or nightmares | 13.6 | 8.5 | 5.1 | 61.6 | 0.51 | 0.762 | |
| Diarrhea | 11.4 | 6.4 | 5.0 | 64 | 0.48 | 0.743 | |
| Muscle aches or pains | 9.1 | 4.3 | 4.8 | 68.1 | 0.42 | 0.743 | |
| Restless feeling in legs | 9.1 | 4.3 | 4.8 | 68.1 | 0.42 | 0.743 | |
| Shortness of breath, gasping for air | 6.8 | 2.1 | 4.7 | 76.2 | 0.35 | 0.718 | |
| Sudden panic or anxiety attacks | 6.8 | 4.3 | 2.6 | 61.6 | 0.67 | 0.848 | |
| Forgetfulness or problems with memory | 6.8 | 4.3 | 2.6 | 61.6 | 0.67 | 0.848 | |
| Agitation | 4.5 | 2.1 | 2.4 | 68.1 | 0.61 | 0.844 | |
| Feeling unreal or detached | 4.5 | 2.1 | 2.4 | 68.1 | 0.61 | 0.844 | |
| Increased saliva in mouth | 2.3 | 0.0 | 2.3 | 100 | 0.48 | 0.743 | |
| Fever | 2.3 | 0.0 | 2.3 | 100 | 0.48 | 0.743 | |
| Burning, numbness, tingling sensations | 2.3 | 0.0 | 2.3 | 100 | 0.48 | 0.743 | |
| Nausea | 13.6 | 12.8 | 0.9 | 51.6 | 1.0 | 1.0 | |
| Unusual tastes or smells | 4.5 | 4.3 | 0.3 | 51.6 | 1.0 | 1.0 | |
| Muscle cramps, spasms, or twitching | 0.0 | 0.0 | 0.0 | NaN | 1.0 | 1.0 | |
| Uncontrollable mouth/tongue  movements | 0.0 | 0.0 | 0.0 | NaN | 1.0 | 1.0 | |
| Stomach cramps | 9.1 | 10.6 | -1.5 | 46.1 | 1.0 | 1.0 | |
| Muscle tension or stiffness | 6.8 | 8.5 | -1.7 | 44.5 | 1.0 | 1.0 | |
| Stomach bloating | 6.8 | 8.5 | -1.7 | 44.5 | 1.0 | 1.0 | |
| Vomiting | 2.3 | 4.3 | -2.0 | 34.8 | 1.0 | 1.0 | |
| Sweating more than usual | 6.8 | 10.6 | -3.8 | 39.1 | 0.72 | 0.879 | |

***Supplementary table 4. DESS symptom incidences.*** *Reported frequencies of each DESS symptom at time point main assessment 2 in %. Patients in the discontinuation group had discontinued their medication, while those in the control group had not. p values based on Fisher’s exact test. Correction for multiple comparisons via fals discovery rate (FDR).*

## Temporal course of depressive and discontinuation symptoms

The following model was used for the fitting, where Outcome ∈ (DESS, IDS):

Outcome ~ negative−binomial (μ,scale)

log(μ) ~ α + β∗time + γ∗time^2^+1|Id

The following vaguely informative priors were used:

α ~ norm(0,5)

β, γ ~ norm(0,1)

scale ~ cauchy(0,2)

## Correlation of IDS and DESS scores

DESS_t_ ~ negative−binomial(μ,scale)

log(μ) ~ α∗DESS_t−1_

log(μ) ~ α∗IDS_t_

The following vaguely informative priors were used for all analyses:

α, β ~ norm(0,1)

scale ~ cauchy(0,2)

Pearson’s correlation between timepoint of main assessment 2 and DESS score at main assessment 2 in discontinuation group: Pearson’s r = -0.41 (95% CI: [-0.63, -0.13], t = -3, df = 42, p-value = 0.005)

## Time course of DESS and IDS

 
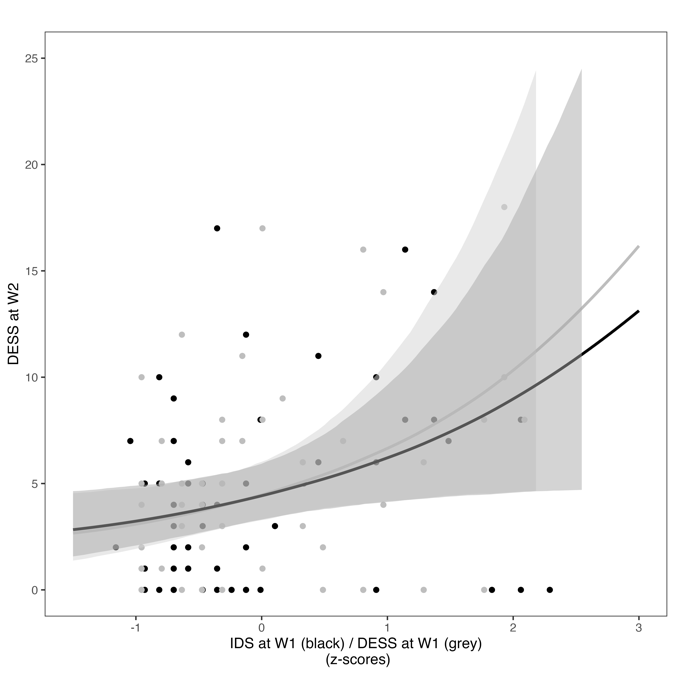

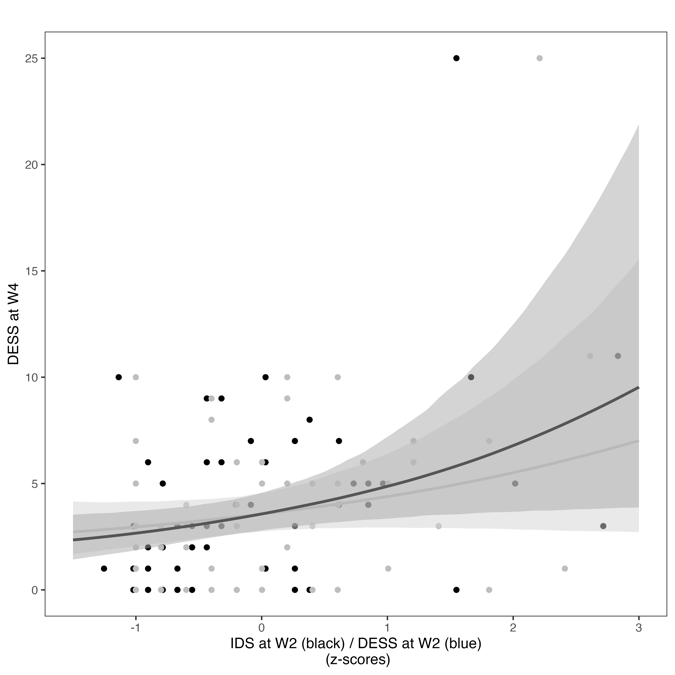


***Supplementary figure 2.*** *Univariate regression with DESS or IDS as predictors and DESS at following assessment as the outcome. Black lines represent the estimated relationship between IDS scores and subsequent DESS scores, whereas grey lines show the association between DESS scores across consecutive assessments. Shaded areas represent 95% posterior intervals of means. Predictors were z-scores to facilitate interpretation of results. W1: week one, W2: week two, W4: week four.*


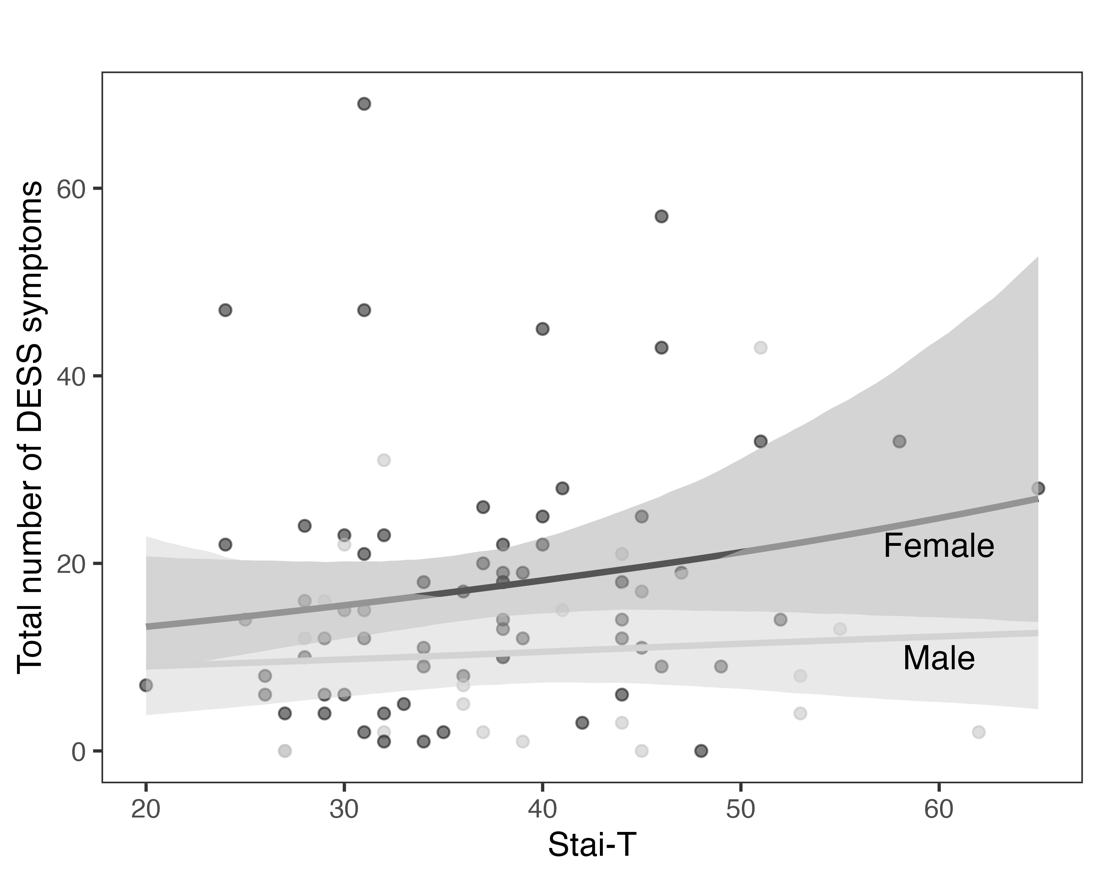


***Supplementary figure 3.*** *Negative-binomial regression with sex, STAI-T and their interaction as predictors. Regression lines are plotted for each sex individually, red areas represent 95% posterior intervals.*

*
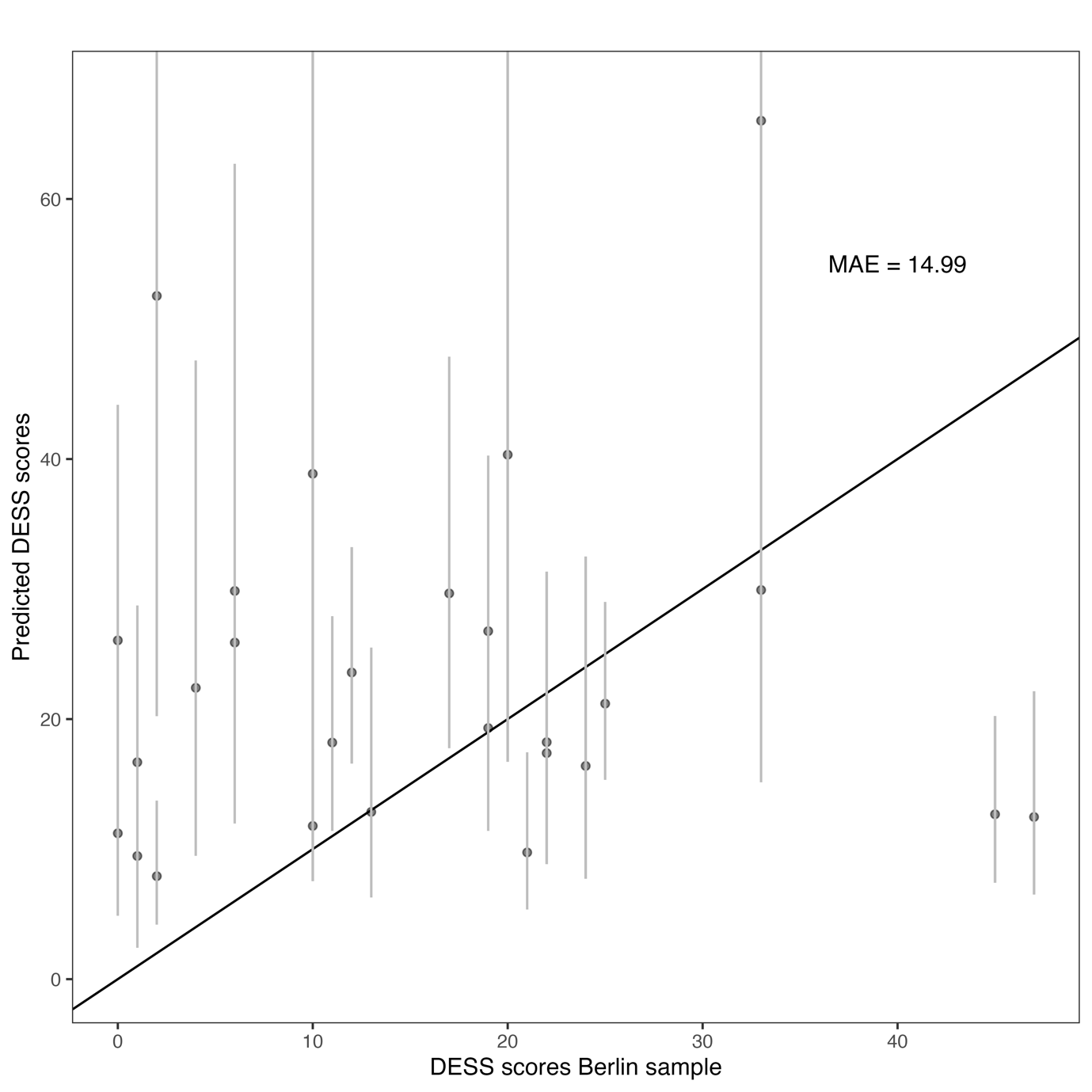
*

**Supplementary figure 4**. Out-of-sample validation. Observed versus predicted number of DESS symptoms after antidepressant discontinuation for patients in the Berlin sample, where the Zurich sample was used for model fitting. Dots represent data points, vertical lines 95% posterior intervals of predicted values.

## Antidepressant type and ADS


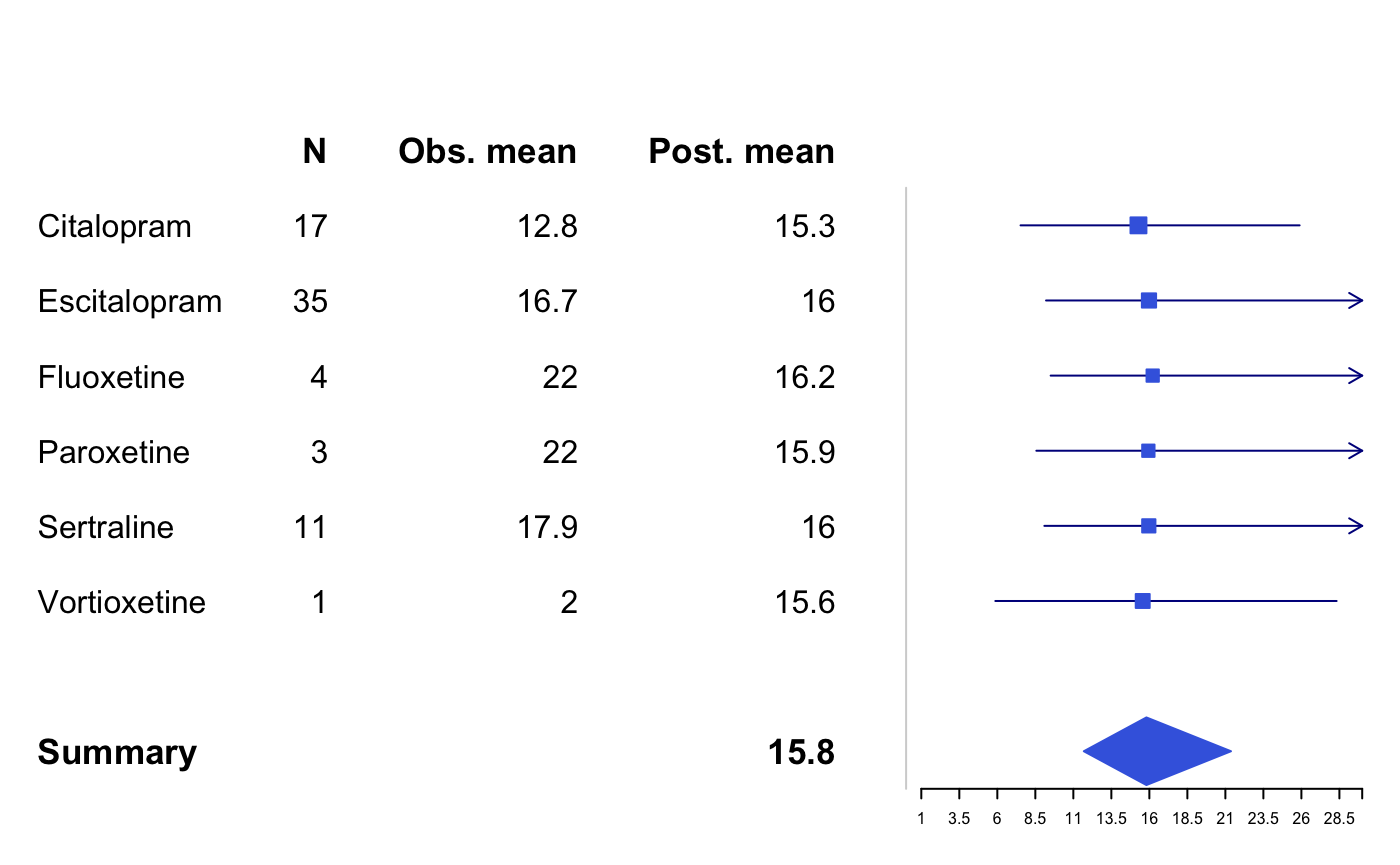


***Supplementary figure 5.*** *Posterior estimates and observed number of withdrawal symptoms for each SSRI antidepressant. Varying intercept regression with antidepressant type nested within antidepressant class.*

## Association of medication and personal factors with ADS

General model:

DESS_total_ ~ negative−binomial(μ,scale)

log(μ) ~ α+ β * predictor

Antidepressant class model:

DESS_total_ ~ negative−binomial(μ,scale)

log(μ) ~ α+ β[ADClass] + γ[ADType:ADClass]

Vaguely informative priors:

α, β ~ norm(0,1)

scale ~ cauchy(0,2)

|  | **Weak prior** | **Strong prior** | **Frequentist** |
| --- | --- | --- | --- |
| Tapering duration (months) | [0.98, 1.26] | [0.98, 1.27] | 0.34 [-0.34, 1.03] |
| Drug half-life (days)  Duration of treatment (years)  Medication load (z-score) | [0.73, 1.46]  [0.94, 1.08]  [0.85, 1.30] | [0.75, 1.47]  [0.94, 1.09]  [0.86, 1.31] | 0.58 [-0.49, 1.65]  -0.05 [-0.41, 0.32]  0.31 [ -0.67, 1.30] |
| Age of Onset (decades) | [0.13, 4.13] | [0.34, 4.98] | -1.02 [-10.54, 8.50] |
| Sex (female)  STAI-T (z-score) | [1.05, 2.52]  [0.92, 1.29] | [1.10, 2.59]  [0.92, 1.32] | -2.37 [-4.64, -0.11]  0.69 [-0.22, 1.59] |

***Supplementary table 5. Association of clinical factors with DESS symptoms.*** *Sensitivity analyses for the association analysis. Posterior mean and 95% posterior intervals for univariate regressions with different predictor. Weak prior: normal(0, 2.5), assumes minimal prior knowledge; strong prior: normal(1, 1) reflects stronger prior belief in a positive association around 1. Frequentist analysis: Average DESS score after discontinuation as outcome. Mean estimate and confidence interval.*

## Prediction of ADS severity


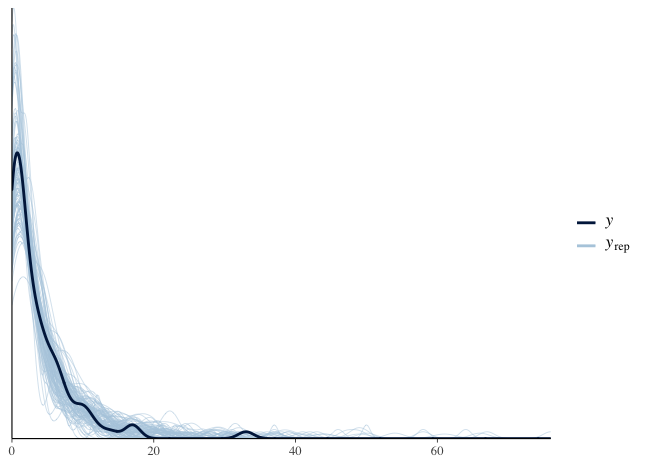


***Supplementary figure 6.*** *Posterior samples from interaction model (DESS + STAI + Interaction) with negative binomial distribution (y_rep_) plotted against observed data (y). This figure shows that the negative binomial distribution is an appropriate assumption for the distribution of DESS scores.*

## Relapse association analysis

| **Variable** | **Bayesian Interval** | **Risk Difference (%)** | **p-value** |
| --- | --- | --- | --- |
| DESS (< 14 days) | 0.50 [-0.06, 1.14] | 12.40 [-1.40, 27.70] | 0.09 |
| DESS (day 15-30) | 0.45 [-0.03, 0.99] | 11.20 [-0.80, 24.20] | 0.09 |
| DESS (somatic only) | 0.47 [-0.01, 1.01] | 11.60 [0.20, 24.80] | 0.07 |
| DESS (excl. early relapse) | 0.51 [0.02, 1.07] | 12.2 [0.0, 26.1] | 0.06 |
| Specific DESS symptoms | 0.50 [-0.03, 1.10] | 11.0 [-0.0, 25.3] | 0.08 |

***Supplementary table 6. Association between DESS scores and relapse risk.*** *Sensitivity analyses. The Bayesian Interval column shows the posterior effect estimates with 95% credible intervals. The Risk Difference (%) column reports the estimated difference in relapse risk percentages, with associated 95% intervals. The p-value column provides the p-value for corresponding Frequentist regression.*

We conducted best-worst, as well as multiple random imputations (coinflip), to address missing values for outcome variable relapse.

| **Outcome** | **Coefficient** | **95% interval** | **p value** |
| --- | --- | --- | --- |
| Complete case | 0.50 | [-0.03, 1.10] | 0.08 |
| Worst | 0.47 | [-0.02, 1.03] | 0.07 |
| Best | 0.38 | [-0.10, 0.90] | 0.13 |

***Supplementary table 7. Association between specific DESS symptoms and relapse risk.*** *Results of Bayesian logistic regression with average of specific DESS symptoms as predictor. Complete case: only patients with relapse status were included. Worst: all patients with missing relapse status were assumed to have relapsed. Best: all patients with missing relapse status were assumed to have not relapsed. P value for corresponding Frequentist analysis.*

|  | **Worst** | **Best** |
| --- | --- | --- |
| STAI-T  IDS  DESS (average) | 0.47 [0.05, 0.91]  0.68 [0.21, 1.18]  0.62 [0.16, 1.20] | 0.34 [-0.10, 0.76]  0.56 [0.12, 1.06]  0.46 [-0.00, 0.98] |
|  |  |  |
|  |  |  |

***Supplementary table 8. Best-worst sensitivity analysis of relapse association analysis****. Logistic regression with relapse as outcome. For 5 missing data points regarding relapse status, the “worst” scenario assumes that all 5 patients relapsed. In the “best” scenario, none of the patients is assumed to have relapsed.*

|  | **% of intervals not including 0** |
| --- | --- |
| STAI-T  IDS  DESS (average) | 32  100  66 |
|  |  |
|  |  |

***Supplementary table 9.*** *Results of multiple random imputations for logistic regression with relapse as outcome. 5 missing relapse values were imputed via “coin flip”, each was run 100 times. Results show percentage of posterior intervals of slope coefficient not including zero.*

## Proportional hazard model

| **Multiple regression** | **Coefficient (95% Interval)** |
| --- | --- |
| Average DESS  Sex  Age  STAI-T  **Multiple regression**  Average IDS  Sex  Age  STAI-T | 1.36 [1.01, 1.83]  1.24 [0.49, 3.13]  1.03 [0.99, 1.06]  1.03 [0.99, 1.07]  **Coefficient (95% Interval)**  1.50 [1.08, 2.08]  1.23 [0.49, 3.07]  1.03 [0.99, 1.06]  1.02 [0.98, 1.05] |

***Supplementary table 10.*** *Results of multiple cox regression. Posterior Mean and 95% intervals. Full case analysis.*

|  | **Worst** | **Best** |  |
| --- | --- | --- | --- |
| STAI-T  IDS  DESS | 0.35 [-0.08, 0.79]  0.55 [0.10, 1.04]  0.44 [-0.01, 0.94] | 0.46 [0.05, 0.90]  0.66 [0.20, 1.18]  0.59 [0.07, 1.13] | |
|  |  |  |  |
|  |  |  |  |

***Supplementary table 11.*** *Sensitivity analyses for univariate cox regressions. Worst: All missing values were replaced with “relapse”. Best: All missing values were replaced with “no relapse”. Posterior Mean and 95% intervals.*
